# *Coptis wawushanensis* (Ranunculaceae), a new species from Sichuan, China

**DOI:** 10.3897/phytokeys.272.162961

**Published:** 2026-03-27

**Authors:** Yang Xiao, Chi Zhang, Wenjia Ke, Zhongxiang Tang, Xin Chen, Zhuyun Yan, Liangke Song, Jue Yang, Wenzhong Shen, Tao Zhou, Yuntong Ma

**Affiliations:** 1 State Key Laboratory of Southwestern Chinese Medicine Resources, Chengdu University of Traditional Chinese Medicine, Chengdu 611137, Sichuan, China Southwest Jiaotong University Chengdu China; 2 College of Pharmacy, Chengdu University of Traditional Chinese Medicine, Chengdu 611137, Sichuan, China College of Pharmacy, Chengdu University of Traditional Chinese Medicine Chengdu China; 3 Southwest Jiaotong University, Chengdu 610031, Sichuan, China State Key Laboratory of Southwestern Chinese Medicine Resources, Chengdu University of Traditional Chinese Medicine Chengdu China; 4 Meishan Ya Lian Engineering Technology Research Center, Meishan 620360, Sichuan, China Meishan Ya Lian Engineering Technology Research Center Meishan China

**Keywords:** China, *Coptis wawushanensis*, Ranunculaceae, Sichuan Province, taxonomy

## Abstract

Based on morphological and plastid data, *Coptis wawushanensis* (Ranunculaceae), a new species from Hongya, Sichuan, China, is described and illustrated. Phylogenetic analyses (Maximum likelihood and Bayesian inference) recover *C. wawushanensis* as sister to *C. huanjiangensis* with strong support, and place them in a well-supported clade including *C. deltoidea* and *C. omeiensis*. Morphologically, the new species most closely resembles *C. omeiensis*, but differs by an ovate leaf blade (vs. lanceolate to narrowly ovate), a central leaf segment 1.5–2.5× as long as the lateral segments (vs. 3–3.5×), shorter scapes (5–8 cm vs. 15–27 cm), and well-developed stolons (vs. absent). It is further distinguished from its sister species *C. huanjiangensis* by leaf blade shape (ovate vs. ovate-triangular), shorter scapes (5–8 cm vs. 20–32 cm), and the presence of stolons (vs. absent). An identification key to the new species and its closest relatives is provided, and its distribution and habitat are documented.

## Introduction

*Coptis* Salisb. (Ranunculaceae) is a perennial herbaceous genus characterized by yellow rhizomes and numerous fibrous roots, comprising approximately 18 species worldwide. It displays a classic East Asian–North American disjunction and occurs predominantly in warm- to cool-temperate coniferous and mixed forests (Xiang et al. 2018). Notable for its abundance of berberine and other bioactive alkaloids, *Coptis* exhibits diverse pharmacological properties and has long been integral to traditional Chinese medicine (Lv et al. 2016; Yang et al. 2017; Wang et al. 2019). It is also documented as a medicinal resource in several other countries (Qi et al. 2019; Pan et al. 2022).

Members of *Coptis* typically possess prominent rhizomes and may produce stolons. Leaves are exclusively basal and borne on elongated petioles, with the lamina distinctly divided into three or five segments. Flowers are solitary or clustered; petals are shorter than sepals and are usually oblanceolate or spatulate. Each flower bears numerous stamens surrounding 8–14 centrally clustered pistils, each distinctly stipitate. Seeds are relatively few, ellipsoid, glossy brown, and marked by subtle longitudinal striations (Fu and Robinson 2001). Traditional taxonomy in the genus relies on a suite of vegetative and reproductive characters, particularly the division and proportions of leaf segments, flower number, the shape and relative length of sepals and petals, and beak length (Xiang et al. 2016). In recent years, systematic surveys and taxonomic revisions in *Coptis* have continued apace in China, with newly reported taxa and local records. In parallel, plastid and other molecular evidence have provided a clearer framework for intrageneric relationships and morphological evolution, facilitating the recognition and description of new taxa (e.g. *C. huanjiangensis* L.Q.Huang, Q.J.Yuan & Y.H.Wang and *C. austrogaoligongensis* C.L.Long & Z.Cheng) (Wang et al. 2022; Cheng et al. 2024).

In China, the diversity of *Coptis* is centered in the Hengduan Mountains, a well-known biodiversity hotspot (Xiang et al. 2018; Sun et al. 2017). The genus *Coptis* in China has been classified into eight species, one variety and one subspecies. Among these, *C. chinensis* Franch., *C. deltoidea* C.Y.Cheng & P.K.Hsiao, *C. omeiensis* (Chen) C.Y.Cheng, *C. teeta* Wall., *C. teeta* subsp. *lohitensis* Pandit & Babu, and the recently described *C. austrogaoligongensis* all occur within the Hengduan Mountains. Most *Coptis* species reproduce sexually through seed production, with dispersal occurring via seeds. An exception is *C. deltoidea*, which propagates obligately via stolon due to its failure to set seed (Li et al. 2010). In contrast, the widespread *C. chinensis*, typically sexual and non-stoloniferous, can facultatively develop stolon-like shoots under environmental stress while maintaining seed production (Pandit and Babu 2003; Wang et al. 2024). Taken together, these observations indicate that the reproductive mode is evolutionarily labile within *Coptis*, raising a testable hypothesis that additional, as-yet-undocumented lineages may combine sexual reproduction with clonal propagation in this region.

During a survey in Heishan Village, Hongya County, Sichuan Province (eastern margin of the Hengduan Mountains), we identified an unrecorded population of *Coptis*. The plants exhibited a distinctive morphological combination, including prominently lobed leaves, extensive stoloniferous growth, and seed production. Subsequent comprehensive morphological and phylogenetic analyses confirmed that this population represents an undescribed species. Herein, we describe and illustrate this distinct species.

## Materials and methods

### Specimen collection and DNA extraction

Specimens and associated DNA samples for this study were collected from the type locality (Heishan Village, Hongya County, Sichuan Province, China) in 2024 and 2025. Voucher specimens were deposited in the herbarium of Chengdu University of Traditional Chinese Medicine (CDCM). The total DNA was extracted from the fresh leaves using the modified CTAB method, and libraries were prepared using the TruePrep DNA Library Prep Kit (NEBNext® Ultra™ II DNA Library Prep, NEB #E7645S/L) (Doyle and Doyle 1987). The plastome sequences of 13 related *Coptis* species (a total of twenty accessions) and two outgroup species [*Asteropyrum peltatum* (Franch.) J.R.Drumm. & Hutch. and *A. cavaleriei* (H.Lév. & Vaniot) J.R.Drumm. & Hutch.] were obtained from GenBank (http://www.ncbi.nlm.nih.gov). Sample information is listed in Suppl. material 1: table SS1.

### Plastome DNA sequencing and assembly

Paired-end sequencing was conducted on a DNBSEQ-T7 platform at the Germplasm Bank of Wild Species in southwest China (Kunming, China). In total, we obtained ~3 GB of data for each sample. Raw reads were checked and filtered using Fastp v.0.24.0 with default parameters (Chen et al. 2018). Plastome sequences from clean data were assembled with GetOrganelle v.1.7.7.1 (Jin et al. 2019). The length and coverage information of the plastid genome (plastome) is shown in Suppl. material 1: table S2. All plastomes were then annotated using PGA v.2.0 with the plastome of *C. quinquesecta* W. T. Wang used as the reference (Zhang et al. 2018, 2025). Annotated plastomes were further manually adjusted in Geneious v.9.0.2 (Kearse et al. 2012). The assembled plastid genome sequences have been deposited in the NCBI GenBank database.

### Phylogenetic tree reconstruction

To determine the phylogenetic position of the new species, we initially retrieved plastome sequences from 13 related *Coptis* species and two outgroup species (*A. peltatum* and *A. cavaleriei* from GenBank (http://www.ncbi.nlm.nih.gov) and NGDC (https://ngdc.cncb.ac.cn). The 79 shared protein-coding genes (PCGs) were extracted using Phylosuite v.1.2.3 (Xiang et al. 2023). Alignments of 79 PCGs and 24 complete plastomes were performed using MAFFT v.7.505 (Katoh and Standley 2013), then manually adjusted utilizing trimAl (Capella-Gutiérrez et al. 2009) and concatenated in Phylosuite v.1.2.3. The optimal partitioning scheme and the best-fit substitution models of the PCGs dataset were estimated using PartitionFinder v.2.1.1 (Lanfear et al. 2016) under the corrected Akaike Information Criterion (AICc) and the greedy algorithm with branch lengths estimated as linked (Suppl. material 1: tables S3, S4). The best-fit substitution models of the complete plastomes dataset were estimated using ModelFinder v.2.2.0 under the Akaike information criterion (AIC) score (Suppl. material 1: table S5) (Kalyaanamoorthy et al. 2017). Phylogenetic analyses were conducted using both Maximum likelihood (ML) and Bayesian inference (BI) approaches in IQ-TREE v.2.2.0 and MrBayes v.3.2.7a, respectively (Ronquist et al. 2012; Nguyen et al. 2015). All analyses were performed in PhyloSuite v.1.2.3. For ML analysis, the 5000 ultrafast bootstraps were employed, with bootstrap iterations set to 1000 (Minh et al. 2013). For BI analysis, the Markov Chain Monte Carlo (MCMC) analyses were run for 10,000,000 generations, in which trees were sampled every 1000 generations with the first 25% discarded as burn-in. Finally, the split frequencies reached an average standard deviation of < 0.01. All phylogenetic trees with bootstrap (BS) values and posterior probabilities (PP) were visualized and edited in FigTree v.1.4.5 (https://github.com/rambaut/figtree).

## Results

### Phylogenetic analysis

**Figure 1.**
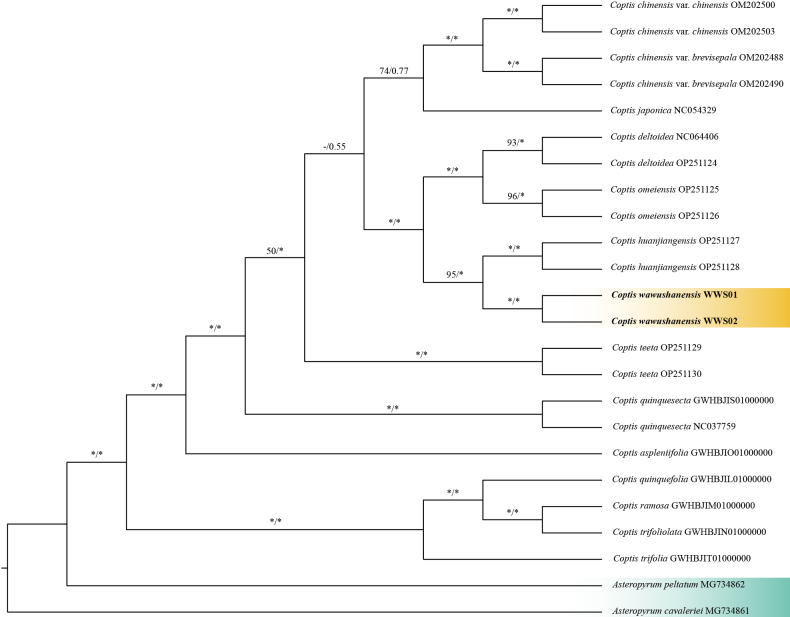
Consensus phylogenetic tree of 79 protein-coding sequences (PCGs) of *Coptis wawushanensis* sp. nov. and related species inferred by maximum likelihood (ML) and Bayesian inference (BI) methods, with *Asteropyrum peltatum* and *A. cavaleriei* as outgroups. Support values are displayed above the branches as ML-BS/BI-PP (“*” indicates BS = 100% or PP = 1.00, “-” indicates BS < 50%).

With two newly sequenced plastomes, phylogenetic trees were constructed based on the two datasets (79 protein-coding sequences and 24 complete plastid sequences), with *Asteropyrum cavaleriei* and *A. peltatum* applied as outgroups. The alignment lengths of the two datasets were 69,486 bp and 154,279 bp; characteristics for the above datasets are listed in Suppl. material 1: table S6. The topologies derived from the two datasets were broadly similar across major clades. Furthermore, the Maximum likelihood (ML) and Bayesian inference (BI) analysis yielded similar results, namely that the two individuals *Coptis wawushanensis* s p. nov. group together, and form a sister clade with *C. huanjiangensis* (Fig. 1 and Suppl. material 2). It is important to note that the resulting phylogeny is solely based on plastome data, and future validation using nuclear data to assess potential cyto-nuclear discordance is essential.

### Taxonomy

#### Coptis wawushanensis

Taxon classificationPlantaeRanunculalesRanunculaceae

Y.T.Ma, T.Zhou & Y.Xiao sp. nov.

FBC0A9E9-43A6-5465-873F-A272FF742A7C

urn:lsid:ipni.org:names:77378087-1

Figs 2, 3

##### Common name.

“瓦屋山黄连” (Wa Wu Shan Huang Lian).

**Figure 2.**
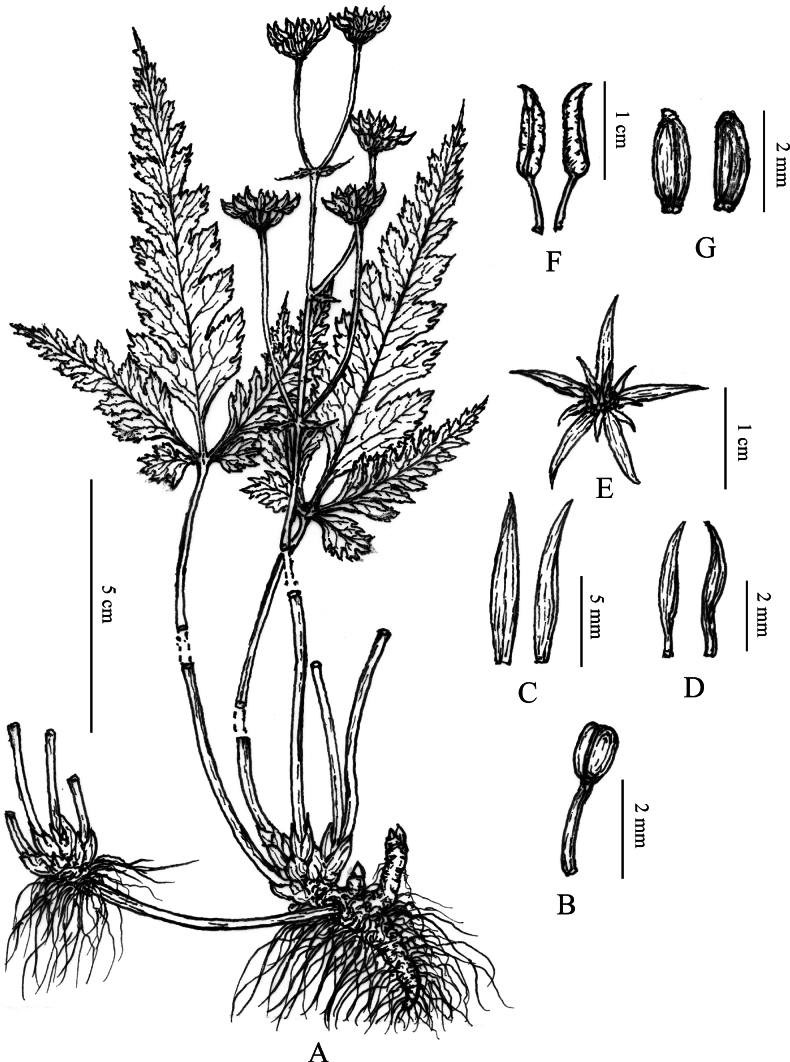
*Coptis wawushanensis* Y.T.Ma, T.Zhou & Y.Xiao, sp. nov. **A**. Habit; **B**. Stamen; **C**. Sepals; **D**. Petals; **E**. Flower; **F**. Follicles; **G**. Seeds. Drawn by Liangke Song.

**Figure 3.**
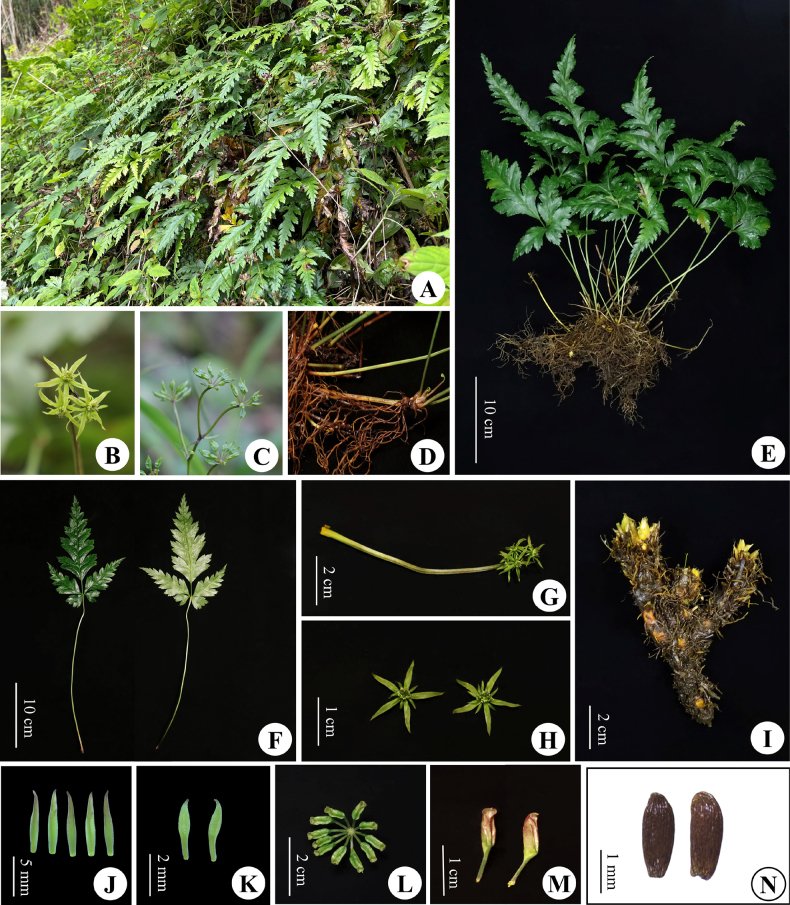
*Coptis wawushanensis* Y.T.Ma, T.Zhou & Y.Xiao, sp. nov. **A**. Species habitat (Heishan Village, Hongya County, Sichuan, China); **B**. Plant in the flowering phase; **C**. Plant in fruiting stage; **D**. Stolon; **E**. Whole plant; **F**. Leaf, frontal and back view; **G**. Inflorescence; **H**. Flowers; **I**. Rhizome; **J**. Sepals; **K**. Petals; **L–N**. Follicles and seeds. Photos by Yang Xiao & Chi Zhang.

##### Type.

• China. Sichuan Province. Hongya County. Heishan Village; 1655 m a.s.l.; 29°28'N, 103°9'E; 16 April 2025; *Yang Xiao & Chi Zhang HS25041601* (holotype: CDCM!) (Suppl. material 3).

##### Diagnosis.

*Coptis wawushanensis* is morphologically similar to *C. omeiensis* but differs by an ovate leaf blade (vs. lanceolate to narrowly ovate), a central leaf segment 1.5–2.5× as long as the lateral segments (vs. 3–3.5×), short scapes 5–8 cm long (vs. 15–27 cm), petals narrowly lanceolate (vs. linear-lanceolate), and well-developed stolons (vs. absent). It differs from its sister species *C. huanjiangensis* by the longer central segment relative to the lateral segments (1.5–2.5× vs. subequal to slightly longer), linear-lanceolate sepals (vs. long-elliptic or lanceolate), narrowly lanceolate petals (vs. spatulate), and stolons present (vs. absent). It can be further distinguished from *C. deltoidea* by leaves deeply 6–11-lobed with remote lobes (vs. 4–6-lobed with lobes ± contiguous), a central leaf segment 1.5–2.5× as long as the lateral segments (vs. only slightly longer), and linear-lanceolate sepals (vs. narrowly ovate).

##### Description.

***Herbs*** perennial. ***Rhizomes*** unbranched or sparsely branched. ***Leaves*** basal, petioles 7–25 cm, glabrous; lamina 7–18 × 4–15 cm, ovate, three-segmented, margins sparsely bearing upturned spinulose hairs; central segment petiolulate, petiolule 0.3–2.5 cm long, 7–16 × 2.5–7 cm, rhombic-lanceolate, deeply 6–11-lobed, lobes remote, ultimate lobes margin acute serrate; lateral segments sessile or with a slender petiolule, petiolule 1–6 mm long, obliquely ovate, unequally bidentate. ***Inflorescences*** cymose, monochasial or dichasial, 3–5-flowered; scape 1–several, erect, 5–8 cm long, glabrous, sulcate; bracts lanceolate, deeply 3-lobed or pectinate-pinnatifid. ***Flowers*** actinomorphic, bisexual; sepals 5, 5.5–9.0 × 0.8–1.6 mm, linear-lanceolate, greenish, sparsely puberulous; petals ca. 10, 2–4 mm long, 1/3–1/2 as long as sepals, narrowly lanceolate, glabrous, apex acuminate; stamens numerous, glabrous, 1.5–3 mm long; pistils 8–12, 2–4 mm long. ***Follicles*** 6.5–10 mm long, stipitate. ***Seeds*** ca. 2 × 0.8 mm, ellipsoid, brown.

##### Distribution and habitat.

Several populations of this species have been found in Wawu Mountain in Meishan City and Emei Mountain in Leshan City, China. It grows in the forest at 1600–2000 m. a. s. l.

##### Etymology.

The specific epithet is derived from the type locality, Wawu Mountain, Sichuan.

##### Phenology.

The species was observed flowering in February–March and fruiting in April–May.

##### Conservation status.

*Coptis wawushanensis* is currently known only from the Wawu Mountain (Meishan City) and Emei Mountain (Leshan City) regions in Sichuan Province, China. Five discrete populations have been documented, each comprising approximately 100 individuals. Pending the collection of more comprehensive data, its conservation status is preliminarily assessed as “Data Deficient (DD)” according to the IUCN guidelines (IUCN Standards and Petitions Committee 2022).

##### Note.

An identification key to *Coptis wawushanensis* and its closest relatives (*C. huanjiangensis*, *C. omeiensis*, and *C. deltoidea*) is provided below.

### Identification key of *Coptis wawushanensis* and its closest species

**Table:** 

1	Sepals linear-lanceolate	**2**
–	Sepals lanceolate, elliptic or narrowly ovate	**3**
2	Leaf blade lanceolate to narrowly ovate; central segment is 3–3.5 times as long as lateral segments; stolons absent	***C. omeiensis***
–	Leaf blade ovate; central segment is 1.5–2.5 times as long as lateral segments; stolons developed	***C. wawushanensis***
3	Stolons absent; leaf margin 4–10 lobed, lobes remote; leaf blade ovate-triangular; central segment similar to or slightly longer than lateral segments; sepals long-elliptic or lanceolate; petals spatulate	***C. huanjiangensis***
–	Stolons developed; leaf margin 4–6 lobed, lobes ± contiguous to each other; leaf blade ovate; central segment slightly longer than lateral segments; sepals narrowly ovate; petals lanceolate	***C. deltoidea***

## Discussion

Leaf and floral traits remain the main basis for species delimitation in *Coptis*, as they are generally stable and readily comparable among taxa (Xiang et al. 2016). Using these characters, we found a consistent set of differences supporting the recognition of *C. wawushanensis* as a distinct species.

Our plastome phylogeny places *C. wawushanensis* as sister to *C. huanjiangensis* with strong support (BS ≥ 95%, PP = 1.0; Fig. 1). This relationship is taxonomically meaningful because it allows direct evaluation of whether the two lineages are morphologically separable. They are, and the differences are concentrated in characters typically treated as diagnostic in the genus, including the length ratio of the central to lateral leaf segments, sepal and petal morphology, and the presence versus absence of stolons (Table 1). The two species are also clearly allopatric: *C. wawushanensis* occurs in the Qionglai Mountains (Wawu Mountain, Meishan City; Emei Mountain, Leshan City), Sichuan (1,600–2,000 m. a. s. l.), whereas *C. huanjiangensis* is restricted to ravines in the Jiuwanshan National Nature Reserve, Huanjiang County, Guangxi (800–1,200 m. a. s. l.) (Wang et al. 2022). The 800 km disjunction further supports geographic and ecological separation (Wu et al. 2023). Plastome data alone cannot fully resolve the speciation history; nuclear loci, broader population sampling, and population genetic analyses will be useful to test the current hypothesis of recent divergence.

**Table 1.** Distinguishing features of *Coptis wawushanensis* in comparison with its closest species.

Characters	*C. wawushanensis*	*C. omeiensis*	*C. huanjiangensis*	*C. deltoidea*
**Leaf blade**	ovate, 7–18 × 4–15 cm, papery to subleathery	lanceolate to narrowly ovate, 6–16 × 3.5–6.3 cm, subleathery	ovate-triangular, 12–22 × 9–22 cm, papery to subleathery	ovate, 4–16 × 5–15 cm, papery to subleathery
**Leaf margin**	deeply 6–11 lobed, lobes remote	7–14 lobed, lobes remote	deeply 4–10 lobed, lobes remote	4–6 lobed, lobes ± contiguous to each other
**Lateral segment length relative to the centra**l	central segment 1.5–2.5 times longer than the lateral segments	central segment 3–3.5 times longer than the lateral segments	central segment similar to or slightly longer than lateral segments	central segment slightly longer than lateral segments
**Petiole length**	7–25 cm	5–14 cm	15–40 cm	6–18 cm
**Scape length**	5–8 cm	15–27 cm	20–32 cm	slightly longer than leaves
**Sepal number**	5	5	5 or 6	5
**Sepal shape**	linear-lanceolate	linear-lanceolate	elliptic or lanceolate	narrowly ovate
**Petal shape**	narrowly lanceolate	linear-lanceolate	spatulate	lanceolate
**Stolons**	Present	Absent	Absent	Present

Within the same well-supported clade, *C. wawushanensis* groups with *C. deltoidea* and *C. omeiensis* (BS = 100%, PP = 1.0). Although *C. wawushanensis* is most similar to *C. omeiensis* in overall appearance, it is readily identified by the diagnostic combination summarized in the species diagnosis (Table 1). It also differs from *C. deltoidea* in several discrete traits (Table 1). Reproductive mode may further separate these taxa: *C. wawushanensis* reproduces by seeds and by stolons; *C. deltoidea* largely relies on stolons because triploidy (2n = 3x = 27) is associated with gamete abortion; and *C. omeiensis* reproduces sexually and lacks stolons (Huang et al. 2013). In combination, the plastome placement, consistent morphology, and geographic isolation justify treating *C. wawushanensis* as an independent species.

## Supplementary Material

XML Treatment for Coptis wawushanensis

## Additional information

### Use of AI

No use of AI was reported.

### Author ORCIDs

Yang Xiao https://orcid.org/0009-0007-5171-1215

Chi Zhang https://orcid.org/0009-0000-5546-2606

Wenjia Ke https://orcid.org/0009-0008-1626-8016

Zhongxiang Tang https://orcid.org/0009-0008-5739-705X

Xin Chen https://orcid.org/0000-0002-7099-4499

Zhuyun Yan https://orcid.org/0000-0003-2501-3652

Liangke Song https://orcid.org/0009-0008-0189-3310

Jue Yang https://orcid.org/0009-0000-6284-6405

Wenzhong Shen https://orcid.org/0009-0001-1256-5055

Tao Zhou https://orcid.org/0009-0004-3142-5906

Yuntong Ma https://orcid.org/0000-0001-6554-5317

### Data availability

All of the data that support the findings of this study are available in the main text or Supplementary Information.

## Supplementary materials

Supplementary material 1 Supplementary tables Data type zip Explanation note **table SS1**. Accession number of 24 sequenced or downloaded plastid genomes in this study. **table S2**. Statistics of plastomes of *Coptis wawushanensis*. **table S3**. Optimal partitioning scheme and best-fit substitution models used for the 79 PCGsML phylogenetic analysis. **table S4**. Optimal partitioning scheme and best-fit substitution models used for the 79 PCGsBI phylogenetic analysis. **table S5**. Best-fit substitution models used for the complete plastid genomes dataset phylogenetic analysis. **table S6**. Statistics of all datasets employed for the phylogenetic analysis of *Coptis*. https://binary.pensoft.net/file/1570978This dataset is made available under the Open Database License (http://opendatacommons.org/licenses/odbl/1.0/). The Open Database License (ODbL) is a license agreement intended to allow users to freely share, modify, and use this Dataset while maintaining this same freedom for others, provided that the original source and author(s) are credited. Yang Xiao

Supplementary material 2 Phylogenetic tree Data type svg Explanation note Consensus phylogenetic tree of complete plastome sequences of *Coptis wawushanensis* sp. nov. and related species inferred by Maximum likelihood (ML) and Bayesian inference (BI) methods, with *Asteropyrum peltatum* and *A. cavaleriei* as outgroups. Support values are displayed above the branches as ML-BS/BI-PP (“*” indicates BS ≥ 95% or PP = 1.00). https://binary.pensoft.net/file/1570979This dataset is made available under the Open Database License (http://opendatacommons.org/licenses/odbl/1.0/). The Open Database License (ODbL) is a license agreement intended to allow users to freely share, modify, and use this Dataset while maintaining this same freedom for others, provided that the original source and author(s) are credited. Yang Xiao

Supplementary material 3 Photo of the holotype Data type jpg Explanation note The photo of the holotype is stored at the CDCM with the accession number HS25041601. https://binary.pensoft.net/file/1570980This dataset is made available under the Open Database License (http://opendatacommons.org/licenses/odbl/1.0/). The Open Database License (ODbL) is a license agreement intended to allow users to freely share, modify, and use this Dataset while maintaining this same freedom for others, provided that the original source and author(s) are credited. Yang Xiao, Chi Zhang, Wenjia Ke, Zhongxiang Tang, Xin Chen, Zhuyun Yan, Liangke Song, Jue Yang, Wenzhong Shen, Tao Zhou, Yuntong Ma
