# Supplementary figures and images for: Coptis wawushanensis (Ranunculaceae), a new species from Sichuan, China
Source: PhytoKeys. 2026 Mar 27;272:107–19. doi: 10.3897/phytokeys.272.162961 (PMC13049450; doi:10.3897/phytokeys.272.162961)

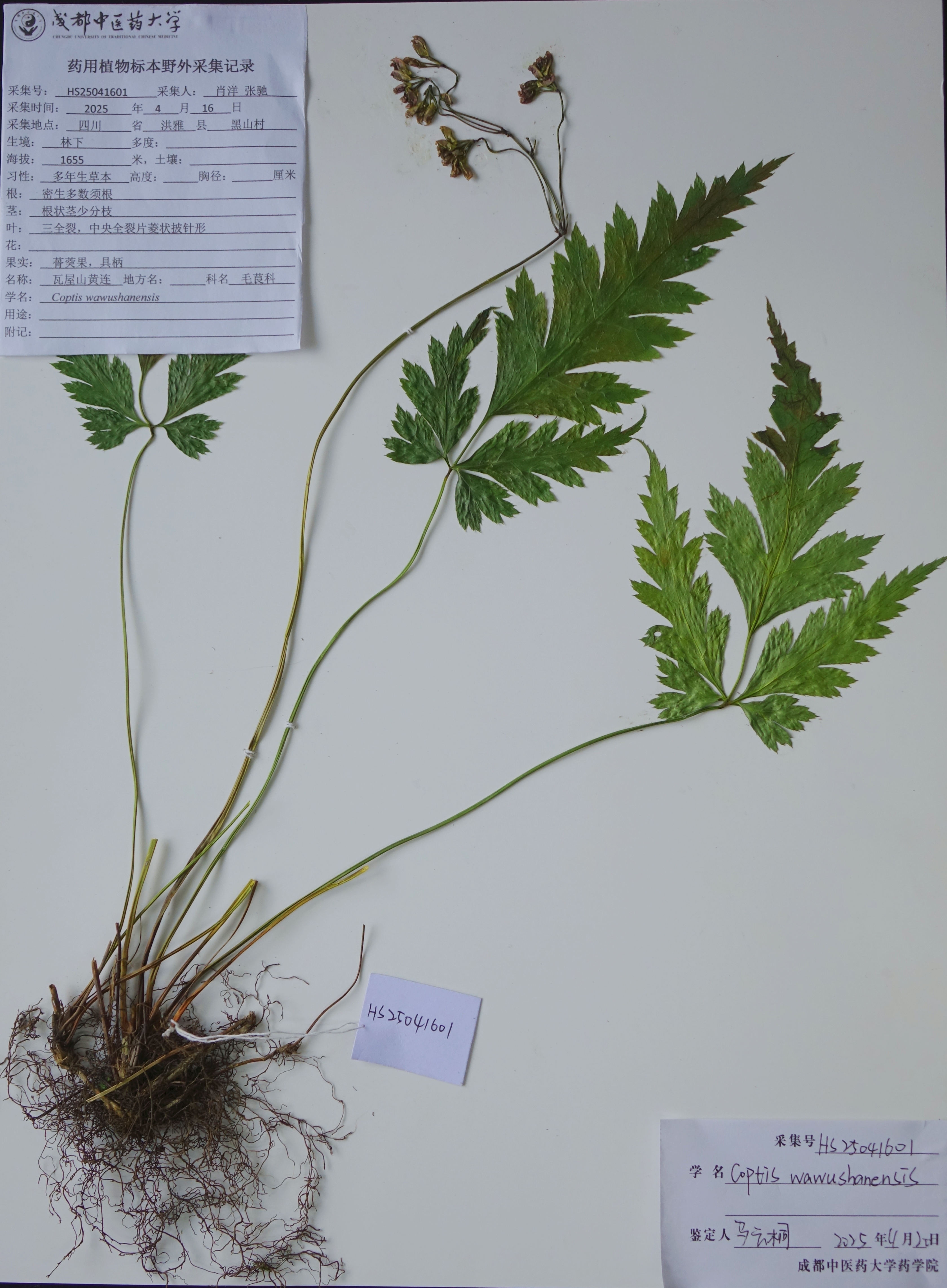

Supplement: Supplementary material 3 — Photo of the holotype [file phytokeys-272-107_article-162961__-s003.jpg]
